# Bisphenol S Alters the Steroidome in the Preovulatory Follicle, Oviduct Fluid and Plasma in Ewes With Contrasted Metabolic Status

**DOI:** 10.3389/fendo.2022.892213

**Published:** 2022-05-24

**Authors:** Ophélie Téteau, Philippe Liere, Antoine Pianos, Alice Desmarchais, Olivier Lasserre, Pascal Papillier, Claire Vignault, Marie-Emilie Lebachelier de la Riviere, Virginie Maillard, Aurélien Binet, Svetlana Uzbekova, Marie Saint-Dizier, Sebastien Elis

**Affiliations:** ^1^CNRS, IFCE, INRAE, Université de Tours, PRC, Nouzilly, France; ^2^U1195 INSERM - Université Paris Saclay, Le Kremlin-Bicêtre Cedex, France; ^3^INRAE, PAO, Nouzilly, France; ^4^Service de Médecine et Biologie de la Reproduction, CHRU de Tours, Tours, France; ^5^Service de Chirurgie pédiatrique viscérale, urologique, plastique et brûlés, CHRU de Tours, Tours, France

**Keywords:** endocrine disruptors, bisphenol S, metabolic status, ewe, steroidome

## Abstract

Bisphenol A (BPA), a plasticizer and endocrine disruptor, has been substituted by bisphenol S (BPS), a structural analogue that had already shown adverse effects on granulosa cell steroidogenesis. The objective of this study was to assess the effect of chronic exposure to BPS, a possible endocrine disruptor, on steroid hormones in the ovary, oviduct and plasma using the ewe as a model. Given the interaction between steroidogenesis and the metabolic status, the BPS effect was tested according to two diet groups. Eighty adult ewes were allotted to restricted (R) and well-fed (WF) groups, that were further subdivided into two subgroups. Ewes were exposed to 50 µg BPS/kg/day in their diet (R50 and WF50 groups) or were unexposed controls (R0 and WF0 groups). After at least 3 months of BPS exposure, preovulatory follicular fluid, oviduct fluid and plasma were collected and steroid hormones were analyzed by gas chromatography coupled with tandem mass spectrometry (GC-MS/MS). A deleterious effect of restricted diet on the volume of oviduct fluid and numbers of pre-ovulatory follicles was observed. Exposure to BPS impaired estradiol concentrations in both follicular and oviduct fluids of well-fed ewes and progesterone, estradiol and estrone concentrations in plasma of restricted ewes. In addition, a significant interaction between metabolic status and BPS exposure was observed for seven steroids, including estradiol. In conclusion, BPS acts in ewes as an endocrine disruptor with differential actions according to metabolic status.

## 1 Introduction

Bisphenol A (BPA), a plasticizer, was reported as an endocrine disruptor that impairs both male and female reproductive function (1–3), and has been banned in the food industry in several countries (Canada, Belgium and France). BPA was then replaced by structural analogues, including bisphenol S (BPS). The use of these plasticizers in food packaging explains why the main source of exposure to bisphenols is through contaminated food (4, 5). However, BPS was detected in human urine and follicular fluid at average concentrations ranging from 2 to 5 nM (6, 7), and has been reported to exhibit harmful effects on health (8). Bisphenols are able to bind to estrogen receptors (9) and are considered as estrogenomimetics. *In vitro* acute exposure of ovarian granulosa cells to BPS impairs progesterone and estradiol secretion in bovine, porcine, human and ovine species with variations according to the culture time point and BPS concentration (6, 10–12). Bisphenols can also bind to corticoid receptors and alter adrenal hormone secretion (2). Furthermore, chronic low exposure of ewes to BPS through the diet has been shown to impair oocyte quality and embryo production *in vitro* (13). However, whether BPS affects steroid hormone synthesis and secretion *in vivo* remains to be explored.

**Figure 1:**
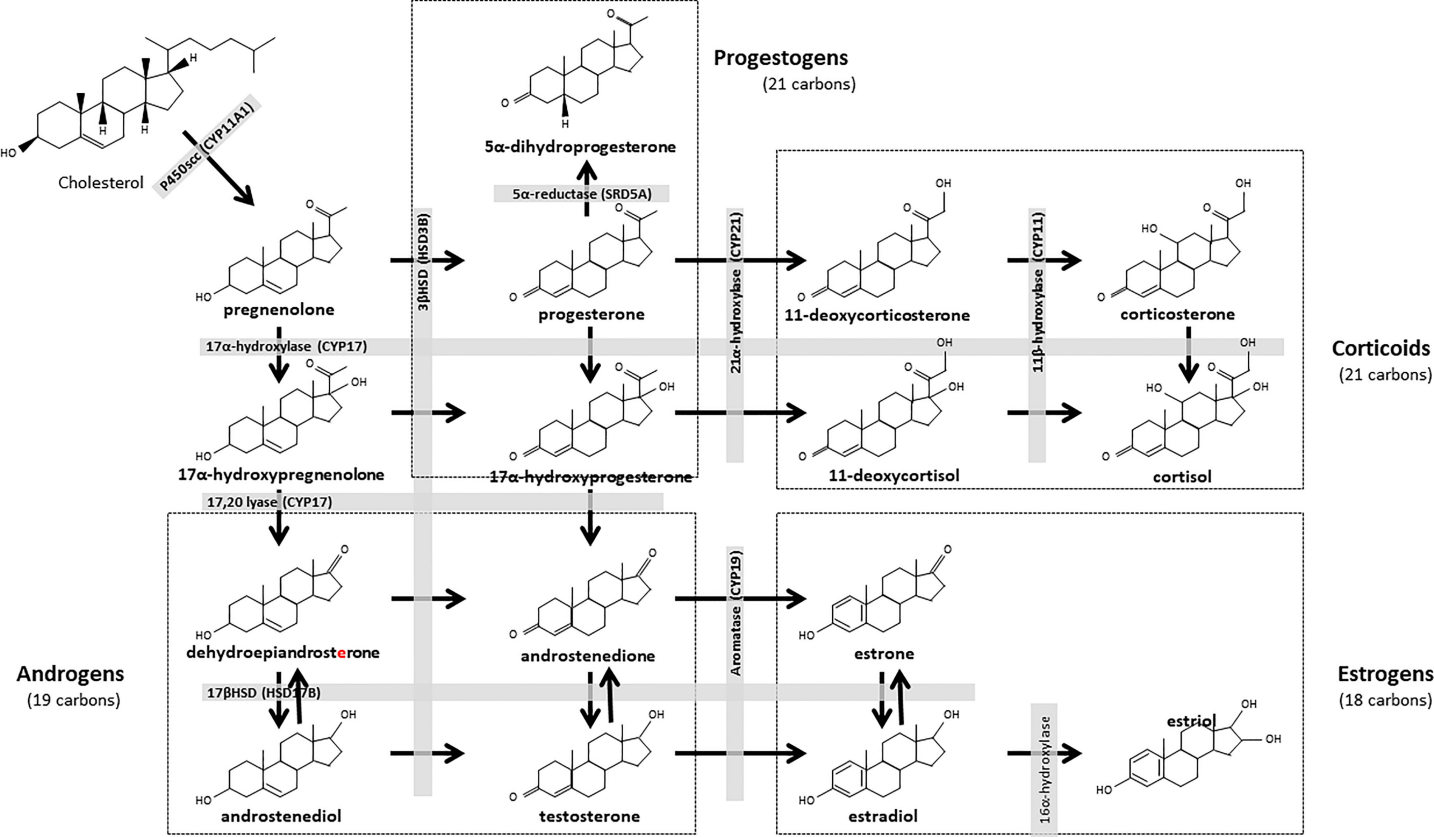
Biosynthesis pathways of steroid hormones. All steroids are derived from cholesterol, which is first converted into pregnenolone and progestogens. Progesterone is the major steroid of the progestogen pathway. This hormone can be converted to androstenedione, then to testosterone. Testosterone is the main steroid in the androgen pathway. This steroid is a precursor to the estrogenic pathway, in which estradiol is the main steroid. The corticoid pathway is derived from the progestogen pathway, with mineralocorticoids (corticosterone) and glucocorticoids (cortisol). CYP, Cytochrome P450 Family; HSD, Hydroxysteroid dehydrogenase; SRD5A, 3-oxo-5α-steroid 4-dehydrogenase.

In mammalian females, the preovulatory follicle is the primary source of sex steroid hormones, which vary in the follicular fluid and circulating plasma according to follicular dynamics throughout the cycle (14, 15). Theca cells synthesize androgens, including testosterone and androstenedione, which are then used by granulosa cells as estrogen (estrone and estradiol) precursors (16, 17). The subsequent increase in estrogen levels leads to the LH surge, which in turn leads to ovulation and to the luteinization of the theca and granulosa layers of the follicle (18). The luteinized follicle and resulting corpus luteum secrete mainly progesterone, which is converted into 5α-dihydroprogesterone (5α-DHP) by ovarian 5α-reductase (19) (**Figure 1**).

Steroid hormones are essential for the development and maturation of the oocyte and the embryo, as well as to establish uterine receptivity required for embryo implantation. Another physiological target of ovarian secreted steroid hormones is the oviduct (also known as the Fallopian tube in humans), which is the site for sperm capacitation, fertilization and early embryo development (20). Steroid hormones are known to regulate the morphology and functions of oviduct cells in mammalian species (21). Among steroid hormones, estrogens and progestogens have been quantified at high levels in the oviduct fluid ipsilateral to ovulation in cows (22). However, to our knowledge, the local concentrations of steroid hormones in other ruminants remain unknown. Furthermore, there is a lack of information on the potential impact of endocrine disruptors like BPS on the oviduct hormonal environment.

Energy metabolism can affect ovarian steroidogenesis and the bioavailability of estrogens and progestogens (23). Lactating cows with high metabolic requirements have less estrogenic dominant follicles compared to non-lactating heifers (24). In sheep, a low body condition score led to a reduction in circulating levels of progesterone and in the ovulation rate (25). Accordingly, an inhibition of lipogenesis in bovine granulosa cells led to a reduction in progesterone secretion *in vitro* (26). Energy metabolism may also interfere with corticoids (27), i.e. adrenal steroids produced from progesterone and 17α-hydroxyprogesterone (**Figure 1**). Cortisol, a glucocorticoid steroid, acts as a catabolic hormone and contributes to the metabolic regulation of reproduction. Cortisol stimulates protein breakdown to release amino acids that can be used in hepatic gluconeogenesis to produce glucose, an energy source used in all reproductive processes (28). Cortisol can increase the penetration of lipids, and simultaneously reduce cholesterol efflux in the blood compartment (29). In the human ovary, intrafollicular levels of cortisol also correlate with cumulus cell lipid content and might regulate cumulus lipolysis (30).

Because metabolic status can regulate steroidogenesis, we hypothesize that the effects of BPS on steroid concentrations could vary according to the metabolic status of the animal, as reported for ovine oocyte quality (13). The objective of the present study was, therefore, to characterize the effects of BPS on all detectable steroid hormones in the preovulatory follicle fluid, oviduct fluid and plasma of adult ewes exhibiting contrasted metabolic status (restricted or well-fed). The ewe is indeed a relevant model species for female reproduction (31, 32) and toxicology studies with bisphenols (33, 34). Eighty ewes underwent both a contrasted diet and at least a three-month period of chronic exposure to BPS through the diet. The steroidome (36 steroids) was then analyzed in the preovulatory follicle fluid, oviduct fluid and plasma using gas chromatography coupled with tandem mass spectrometry (GC-MS/MS), at the preovulatory stage of the cycle, a critical stage characterized by the increased estradiol secretion before the LH surge and ovulation.

## 2 Materials and Methods

### 2.1 Ethics

All experimental procedures were conducted in accordance with the European Directive 2010/63/EU on the protection of animals used for scientific purposes and approved by the French Ministry of National Education, Higher Education, Research and Innovation after ethical assessment by the local ethics committee. “Comité d’Ethique en Expérimentation Animale Val de Loire (CEEA VdL)” (protocols registered under APAFIS numbers 13965-2018042008519239v2 and 14014-2018030717477406v2).

### 2.2 Experimental Design

For two years (2018 and 2019), primiparous Ile-de-France ewes (n = 80; age mean 2.55 ± 0.04 years at the beginning of the experiment) were housed in a sheepfold in batches of 10 ewes. All ewes in one batch were free to interact with each other. Contact with rams and their odors was precluded to avoid any induction of estrus by the male (male effect) during the study.

In June, i.e. at least 3 months before sample collection, ewes with similar age, body weight (BW) and body condition score (BCS) were allotted into two diet groups, as previously described (13). Briefly, the diet consisted of straw added with dietary supplement (AXEREAL Elevage, saint Germain de salles, France) composed of wheat (60%), alfalfa, sugar cane treacle and vitamins with nutritional values of 1.5 Mcal of net energy and 72 g of metabolisable protein per kilogram of dry matter and straw *ad libitum*. One restricted group (R group; n = 40) received 50% of their daily food requirement (0.15 kg of feed per animal) until the target BCS was reached and then 80% of their daily food requirement until the end of the experiment and one well-fed group (WF group; n = 40) received 165% of their daily food requirement until the end of the experiment. The diet was designed according to the INRAE recommendations for the growth and maintenance needs of adult, non-pregnant ewes (35). Briefly, the diet consisted in varying the quantity of a wheat-based food supplement (Agneau-echange, AXEREAL Elevage, Saint Germain de Salles, France). The food supplement was distributed in the morning and the animals had free access to straw and water and minerals to lick. The BW (kg) and BCS (on a scale of 1 to 5, 1 corresponding to very skinny ewes and 5 to very fat ewes) of animals were monitored once a month.

**Figure 2:**
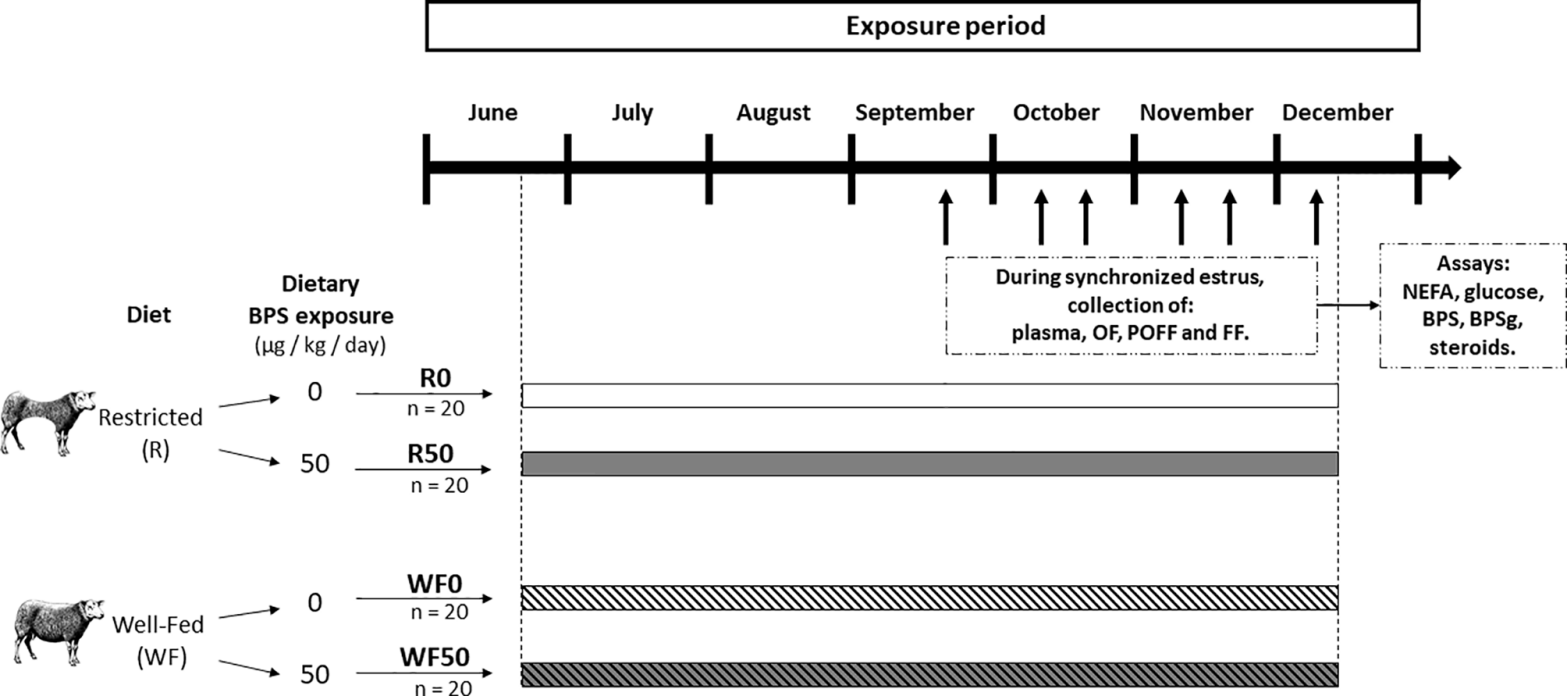
Experimental design. A total of 80 ewes were allocated to one of two diet groups (Restricted, R and Well-Fed, WF), and, in each group, in one of two exposure subgroups (0 or 50 µg BPS/kg/day). Thus, four experimental groups of ewes were formed: restricted without BPS (R0, n = 20), restricted with 50 µg/kg/d of BPS (R50, n = 20), well-fed without BPS (WF0, n = 20), and well-fed with 50 µg/kg/d of BPS (WF50, n = 20). The diet and BPS exposure lasted for at least 3 months before the start of plasma/fluid collection. Between September and December, the estrous cycle of ewes were sequentially synchronized (batches of 6-8 ewes, represented by black arrows), and animals were slaughtered 2 days after PMSG administration for collection of plasma, oviductal fluid (OF), and preovulatory follicular fluid (POFF, diameter ≥ 6 mm), and follicular fluid (FF, < 6mm follicles, n = 20, 5 ewes per experimental groups). NEFA: non-esterified fatty acids.

The restricted and well-fed diet groups were divided into two subgroups according to their dietary exposure to BPS, giving rise to four experimental groups: R0 and WF0 as not exposed controls, and R50 and WF50 exposed to 50 µg BPS/kg/day through the diet (n = 20 per group) (**Figure 2**). This dose was set according to the guidelines established for BPA in Europe in 2015 (36).

Between September and December, i.e. during the natural breeding season, all 80 ewes were synchronized for estrus in batches of 6-8 ewes (including 1-2 ewes per experimental group) with a vaginal progesterone sponge (Chrono-Gest^®^ 20 mg, MSD, Beaucouze, France) for 11 days, followed by an intramuscular administration of PMSG (Synchro-Part^®^ PMSG 400 UI, CEVA Santé Animale, Libourne, France) to induce final follicular growth. Two days after PMSG administration, at the presumptive preovulatory time, ewes were culled by electronarcose and bleeding in the local INRAE experimental slaughterhouse and biological fluids were collected within 15 min after death (**Figure 2**).

### 2.3 Collection and Preparation of Biological Fluids

Blood samples (5 mL) were collected at the time of slaughterhouse bleeding in heparinized tubes (17 IU/mL sodium heparin, Vacutainer^®^; Becton Dickinson and Company, Le Pont de Claix, France). After centrifugation (3,700 g for 30 min at 4°C), plasma were stored at -20°C.

Both ovaries and the genital tract of individual ewes were recovered and immediately processed for collection of biological fluids. Animals with ovarian cysts or apparent pathology in the genital tract were excluded from the study (n = 2), therefore, 78 ewes were used for fluid collection. The preovulatory follicle (diameter ≥ 6 mm) were punctured and the follicular fluid was isolated from granulosa cells by centrifugation (9,600 g for 3 min, 4°C) and then stored at -80°C. The oviducts were collected according to the side of ovulation: in case of bilateral preovulatory follicle, both oviducts were collected, whereas in mono-ovular females, only the oviduct ipsilateral to the preovulatory follicle was collected. The oviducts were cleaned of surrounding tissue and, after elimination of the infundibulum, the content of the ampulla and isthmus was collected by gentle squeezing with a glass slide. The resulting pellet was collected in a 1.5 mL tube and stored on ice before centrifugation. Then, the oviduct fluid was separated from cells by centrifugation (12,000 g for 15 min, 4°C). The volume of the supernatant was measured by pipetting (range: 2-30 µL) and the oviduct fluid was stored at -80°C.

Preliminary experiments showed that 50 µL was the minimal volume required for the detection by GC-MS/MS of estrogens and progestogens in ovine follicular and oviduct fluids at estrus (data not shown). After all collection sessions, samples were thawed on ice for pooling. On the basis of the limited volume of each ewe individual samples, pools of 3-4 ewes were made to reach a final volume of 50 to 60 µL of oviduct or follicular fluids per pool and totals of 4 to 6 biological replicates (pools of ewes) per experimental group (R0, n = 4; R50, n = 4; WF0, n = 6; WF50, n = 5). The same animals were pooled together for plasma (500 µL per pool), oviduct fluid and preovulatory follicle so that results could be compared between compartments. In the following, unless otherwise mentioned, the term “sample” will refer to a pool of biological fluids. The samples were stored at -80°C until steroidomic analysis.

### 2.4 Plasma Glucose and Non-Esterified Fatty Acids (NEFA) Assays

Plasma NEFA and glucose of individual ewes (n = 80) were quantified on a 5 and 2 µL undiluted plasma sample, respectively, by colorimetric enzymatic methods using a Konelab 20 analyzer (Thermo Scientific, Gometz le Châtel, France) and kits provided by Bio-Mérieux (Marcy-l’Etoile, France) and Thermo Scientific (Villebon sur Yvette, France), as previously described (37).

### 2.5 Plasma and Follicular Fluid BPS and BPS-Glucuronide (BPS-g) Assays

Plasma (n = 80) and follicular fluid (< 6 mm follicles, therefore, not the preovulatory follicles, n = 20, 5 ewes per experimental groups) BPS and BPS-g concentrations of individual ewes were determined as previously described (13). Briefly, BPS and BPS-g concentrations were quantified using liquid chromatography-mass spectrometry with an Acquity U-HPLC device coupled to a Xevo-TQ triple quadrupole mass spectrometer (Waters, Saint-Quentin-en-Yvelines, France) operated with positive electrospray ionization and MRM mode. All samples were quantified on the same day. The limit of quantification (LOQ) was set at 0.5 ng/mL (2 nM) for BPS and 0.05 ng/mL (0.10 nM) for BPS-g.

### 2.6 Steroid Measurements in Plasma, Oviduct and Follicular Fluids by GC-MS/MS

#### 2.6.1 Steroid Extraction

Steroids were extracted from plasma, oviduct and follicular fluids with methanol, and the following internal standards were introduced into each sample for steroid quantification: 1 ng of ^13^C_3_-testosterone, ^13^C_3_-17β-E2 (estradiol), ^13^C_3_-estrone, ^13^C_3_-estriol, ^2^H_5_-2-methoxyE2 and ^13^C_3_-androstenedione (IsoSciences, King of Prussia, PA, USA), 2 ng of ^13^C_3_-17α-hydroxyPROG (progesterone), ^13^C_5_-5α-dihydroPROG, epietiocholanolone (Steraloids, Newport, RI, USA), ^13^C_5_-20α-dihydroPROG, ^13^C_3_-PROG, 10 ng of ^2^H_8_-corticosterone and 25 ng of ^13^C_3_-cortisol (CDN Isotopes, Sainte Foy la Grande, France) (**Table 1**).

**Table 1:** Steroids analyzed by gas chromatography coupled to tandem mass spectrometry in ovine plasma, oviduct fluid and follicular fluid.

Steroids	Abbreviation	Internal standards (IS)	IS Quantity (ng)
Pregnenolone	PREG	3β5β-THA	2
20α-dihydropregnenolone	20α-DHPREG	3β5β-THA	2
17α-hydroxypregnenolone	17α-OHPREG	^13^C_3_-17α-OH PROG	2
**Progestogens (C21)**			
Progesterone	PROG (P)	^13^C_3_-PROG	2
5α-dihydroprogesterone	5α-DHP	^13^C_5_-5α-DHP	2
5β-dihydroprogesterone	5β-DHP	^13^C_5_-5α-DHP	2
20α-dihydroprogesterone	20α-DHP	^13^C_5_-20α-DHP	2
3α5α-tetrahydroprogesterone	3α5α-THP	3β5β-THA	2
3α5β-tetrahydroprogesterone	3α5β-THP	3β5β-THA	2
3β5α-tetrahydroprogesterone	3β5α-THP	3β5β-THA	2
3α5α20α-hexahydroprogesterone	3α5α20α-HHP	3β5β-THA	2
3α5β20α-hexahydroprogesterone	3α5β20α-HHP	3β5β-THA	2
3α5β20β-hexahydroprogesterone	3α5β20β-HHP	3β5β-THA	2
3β5α20β-hexahydroprogesterone	3β5α20β-HHP	3β5β-THA	2
3β5α20α-hexahydroprogesterone	3β5α20α-HHP	3β5β-THA	2
17α-hydroxyprogesterone	17α-OHP	^13^C_3_-17α-OH PROG	2
16α-hydroxyprogesterone	16α-OHP	^13^C_3_-17α-OH PROG	2
**Androgens (C19)**			
Testosterone	T	^13^C_3_-T	1
5α-dihydrotestosterone	5α-DHT	3β5β-THA	2
5β-dihydrotestosterone	5β-DHT	3β5β-THA	2
Dehydroepiandrosterone	DHEA	3β5β-THA	2
Androstenediol	ADIOL	3β5β-THA	2
Androstenedione	ADIONE	^13^C_3_-Androstenedione	1
5α-dihydoandrostenedione	5α-DHADIONE	^13^C_5_-5α-DHP	2
**Estrogens (C18)**			
Estradiol	E2	^13^C_3_-17β-E2	1
Estrone	E1	^13^C_3_-E1	1
Estriol	E3	^13^C_3_-E3	1
2-methoxyestradiol	2-ME2	^2^H_5_-2-ME	1
2-hydroxyestradiol	2-OH-E2	^2^H_5_-2-ME	1
**Corticoids (C21)**			
**Glucocorticoids**			
Cortisol	F	^13^C_3_-cortisol	25
Cortisone	E	^13^C_3_-cortisol	25
11-deoxycortisol	11-deoxyF	^13^C_3_-cortisol	25
3α5β-tetrahydrocortisol	3α5β-THF	3β5β-THA	2
**Mineralocorticoids**			
Corticosterone	B	^2^H_8_-B	10
11-dehydrocorticosterone	11-dehydroB	^2^H_8_-B	10
3α5α-tetrahydrodeoxycorticosterone	3α5α-THDOC	3β5β-THA	2

The abbreviations of all detected steroids are given. The identity and the amount of the internal standard are described for each measured steroid.

#### 2.6.2 Steroid Purification

The samples were purified and fractionated by solid phase extraction as previously described (38). Briefly, the extracts were dissolved in 1 mL of methanol and applied to the C18 cartridge (500 mg, 6 mL; International Sorbent Technology, Ystrad Mynach, United Kingdom), followed by 5 mL of methanol/H_2_O (85/15). The flow-through, containing the unconjugated steroids, was collected and dried. After re-conditioning of the same cartridge with 5 mL methanol/H_2_O (2/8), the dried samples were dissolved in methanol/H_2_O (2/8) and re-applied. The cartridge was then washed with 5 mL H_2_O and 5 mL methanol H_2_O (1/1), and unconjugated steroids were eluted with 5 mL methanol/H_2_O (9/1).

The unconjugated steroid-containing fraction was then filtered and further purified and fractionated by high performance liquid chromatography (HPLC). The HPLC system was composed of a WPS-3000SL analytical autosampler and an LPG-3400SD quaternary pump gradient coupled with a SR-3000 fraction collector (Thermo Scientific, San Jose, CA, USA). HPLC separation was achieved with a Lichrosorb Diol column (25 cm, 4.6 mm, 5 mm) in a thermostat block at 30°C. The column was equilibrated in a solvent system of 90% heptane and 10% of a mixture composed of heptane/isopropanol (85/15). Elution was performed at a flow rate of 1 mL/min, with first 90% heptane and 10% of heptane/isopropanol (85/15) for 15 min and then with a linear gradient to 100% acetone in 2 min. This mobile phase was kept constant for 13 min. Two fractions were collected from the HPLC system: 5α/β-dihydroprogesterone (5α/β-DHP) and 5α-dihydroandrostenedione (5α/β-DHADIONE) were eluted in the first HPLC fraction (3-16 min) and was next silylated with 50 µL MSTFA (N-methyl-N-trimethylsilyltrifluoroacetamide)/ammonium iodide (NH_4_I)/dithioerythritol (1000:2:5 vol/wt/wt) for 15 min at 70°C. The second fraction (16-29 min) containing all other targeted steroids was derivatized with 25 µL heptafluorobutyric anhydride and 25 µL anhydrous acetone for 1 h at room temperature. All the fractions were dried under a stream of N_2_ and resuspended in heptane for GC-MS/MS analysis.

#### 2.6.3 Gas Chromatography Coupled With Tandem Mass Spectrometry (GC-MS/MS)

GC-MS/MS analysis of the fluid extracts was performed using an AI 1310 autosampler, a Trace 1310 GC, and a TSQ 8000 tandem mass spectrometer MS/MS (Thermo Scientific) using argon as the collision gas. The injection was performed in splitless mode at 250°C (1 min of splitless time) and the temperature of the gas chromatograph oven was initially maintained at 80°C for 1 min and ramped between 80°C and 200°C at 20°C/min, then ramped to 300°C at 10°C/min, and finally ramped to 350°C at 30°C/min. The helium carrier gas flow was maintained constant at 1 mL/min during the analysis. The transfer line and ionization chamber temperatures were 330°C and 200°C, respectively. Electron impact ionization was used for mass spectrometry with ionization energy of 70eV. GC-MS/MS signals were evaluated using a computer workstation by means of Excalibur software, release 3.0 (Thermo Scientific). The identification of steroids was support by their retention time and according to two or three transitions. Quantification was performed according to the transition giving a signal characterized by the highest signal-to-noise ratio, with a previously established calibration curve (22, 39).

The analytical protocol was validated for all the targeted steroids by using extracts of 200 mg from a pool of male mouse plasma. The evaluation included the limit of detection, linearity, accuracy, as well as intra-assay and inter-assay precision. The limit of detection was determined as the lowest amount of compounds that can be measured by GC-MS/MS with a signal-to-noise ratio ≥3 and ranged from 0.5 to 10 pg/mL. The linearity was assessed by analyzing increasing amounts of mouse plasma extracts (25, 50, 100, and 200 µL) in triplicate. The linearity was satisfactory for all the steroids with a coefficient of correlation ranging from 0.990 to 0.997. The accuracy of the assay was evaluated by determining the analytical recovery, which was defined as *C*/(*C*0+S)x100(%), where *C* is the concentration of the steroid in the spiked plasma extract (100 µL), *C*0 is the concentration of a steroid in the unspiked plasma extract (100 µL) and *S* is the spiked concentration. The accuracy was in the range 95-106%. The coefficient of variation of the intra assay and inter assay was evaluated by analyzing five replicates of 200 µL of plasma extracts and was 7.5% and 10.5%, respectively (40).

All the steroids detected in at least one biological fluid of ewes are described in **Table 1**.

### 2.7 Measurement of Estradiol and Progesterone in Follicular Fluid by Enzymatic Assays

In addition to the steroidomic approach, estradiol and progesterone concentrations were individually measured in 80 preovulatory follicular fluids (n = 20 per group). All measurements were performed during the same experiment. Estradiol was measured using an enzyme immunoassay kit (E2-EASIA-kit, DIAsource, Louvain- La-Neuve, Belgium), according to the manufacturer’s instructions. Estradiol concentrations ranged from 1.56 to 50 pg/mL and the intra-assay coefficient of variation (CV) averaged 8%. Progesterone was measured using an enzyme immunoassay protocol, as described previously (41). Progesterone concentrations ranged from 0.2 to 10 ng/mL and the intra-assay CV was 5.7%.

### 2.8 Statistical Analysis

Statistical analyses were performed using R software (version 3.5.1). The BW (kg), BCS (units) and concentrations of glucose (mg/L), NEFA (µmol/L), BPS and BPS-g (nM), and steroid hormones (ng/mL) are expressed as means ± SEM. The effects of diet, BPS and the interaction between diet and BPS exposure were assessed on follicle size, oviduct fluid volume and BPS, BPS-g and steroid concentrations by the non-parametric two-way ANOVA test (R package lmperm) followed, when significant, by the Tukey *post-hoc* test (R package nparcomp). Differences were considered significant when the *P* value was < 0.05.

## 3 Results

### 3.1. Validation of the Diet Model

The body weight (BW) and body condition score (BCS) of ewes significantly differed between the well-fed (WF) and restricted (R) groups (**Table 2**). The mean BW and BCS of well-fed ewes were higher compared to restricted ewes with average differences of 11 kg and 1 BCS point between groups (p < 0.001). A BPS effect on BCS was found (p = 0.026). Despite being overfed (165% of their energy maintenance needs), the ewes from the WF group did not become overweight. Furthermore, the well-fed diet increased plasma glucose (661.3 ± 16.1 *vs*. 825.2 ± 31.6 mg/L; p < 0.001) and NEFA (152.6 ± 12.7 *vs*. 216.9 ± 23.0 µmol/L; p = 0.018) concentrations in ewes compared to the restricted diet (**Table 2**).

**Table 2:** Characterization of ewes at the time of sample collection.

Parameters	Mean ± SEM				p-value		
	R0	R50	WF0	WF50	Diet effect	BPS effect	Diet x BPS effect
**Diet effect**							
Body weight (kg)	56.06 ± 1.01**^a^**	55.41 ± 1.23**^a^**	67.21 ± 1.14**^b^**	66.28 ± 1.24**^b^**	**< 0.001**	0.509	0.896
Body condition score	2.16 ± 0.08**^a^**	2.21 ± 0.07**^a^**	2.96 ± 0.07**^b^**	3.26 ± 0.09**^b^**	**< 0.001**	**0.026**	0.102
Plasma glucose (mg/L)	638.93 ± 17.61**^a^**	683.65 ± 26.54**^ac^**	786.42 ± 40.89**^bc^**	863.93 ± 47.58**^b^**	**< 0.001**	0.085	0.620
Plasma NEFA (µmol/L)	132.40 ± 14.51**^a^**	172.71 ± 20.25**^ab^**	245.06 ± 37.44**^b^**	181.66 ± 19.99**^ab^**	**0.018**	1.000	**0.044**
**BPS exposure**							
Plasma BPS (nM)	0.00 ± 0.00**^a^**	2.17 ± 0.62**^b^**	0.00 ± 0.00**^a^**	1.98 ± 0.69**^b^**	0.983	**< 0.001**	0.892
Plasma BPS-g (nM)	0.00 ± 0.00**^a^**	193.75 ± 20.09**^b^**	0.00 ± 0.00**^a^**	196.68 ± 22.46**^b^**	1.000	**< 0.001**	1.000
Follicular fluid BPS-g (nM)	0.00 ± 0.00**^a^**	102.51 ± 7.72**^b^**	0.00 ± 0.00**^a^**	43.62 ± 20.60**^b^**	**0.039**	**< 0.001**	**0.038**
**Reproductive function**							
Ovary weight (g)	2.27 ± 0.13	2.36 ± 0.16	2.61 ± 0.14	2.58 ± 0.11	0.051	0.850	0.602
Oviduct fluid volume (µL)	20.92 ± 3.26**^a^**	22.75 ± 2.88**^ab^**	35.82 ± 5.07**^b^**	29.25 ± 7.28**^ab^**	**0.021**	0.594	0.364
Number of follicles: 2 ≥ F > 6 mm	18.60 ± 1.71	22.74 ± 2.36	19.75 ± 2.07	19.26 ± 1.56	0.570	0.387	0.230
Number of follicles: F ≥ 6 mm	1.30 ± 0.15**^a^**	1.42 ± 0.16**^ab^**	1.80 ± 0.12**^b^**	1.63 ± 0.19**^ab^**	**0.031**	0.876	0.413

Ewes were either restricted (R groups) or well-fed (WF groups) and received BPS 0 or 50 µg/kg/day, for at least three months before the start of biological fluid collection. Thus, four groups were formed: R0 (n = 20), R50 (n = 20), WF0 (n = 20) and WF50 (n = 20). Results are presented as mean ± SEM. Tukey post-tests are indicated by letters and values with different letters are significantly different (p < 0.05). Two-way ANOVA p-values are presented for the effects of diet, dietary exposure to BPS and the interaction of these effects. Bold text indicates significant differences (p < 0.05).

### 3.2 BPS and Its Metabolite Were Detected in the Plasma and Follicular Fluid of Exposed Ewes

Ewes were exposed to BPS for on average 5.4 ± 0.1 months (range: 3.6-7.9 months) in the R50 and WF50 groups before sample collection. In these groups, BPS and BPS-g, a marker of internal BPS exposure, were assessed in the plasma of all ewes (1h30 in average after the last exposure) and in the follicular fluid (< 6 mm follicles, therefore not from the preovulatory follicle) of 20 randomly chosen ewes (n = 5 R0, 5 R50, 5 W0 and 5 WF50) at the time of slaughter (therefore 24 h in average after the last exposure. BPS and BPS-g were detected in the plasma, with no difference according to metabolic status (**Table 2**). In the follicular fluid of < 6 mm follicles (not preovulatory follicles), BPS-g was detectable in all ewes, and was affected by metabolic status (p = 0.039; **Table 2**). BPS was detectable in the follicular fluid of only one R50 ewe among the five R50 and five WF50 ewes. As expected, both BPS and BPS-g were undetectable in the plasma and follicular fluid of control unexposed ewes.

### 3.3 Well-Fed Ewes Had Increased Numbers of Preovulatory Follicles and Volume of Oviduct Fluid

The well-fed diet tended to increase the average weight of both ovaries (2.31 ± 0.10 g *vs*. 2.59 ± 0.09 g for R and WF ewes, respectively; p = 0.051) and increased the number of preovulatory follicles (1.36 ± 0.11 *vs*. 1.72 ± 0.11, respectively; p = 0.031; **Table 2**). Moreover, the volume of the oviduct fluid was significantly greater in well-fed compared to restricted ewes (33.1 ± 4.2 µL *vs*. 21.8 ± 2.2 µL, respectively; p = 0.021; **Table 2**).

### 3.4 Exposure to BPS and Metabolic Status Altered the Steroid Content in the Preovulatory Follicle, Oviduct Fluid and Plasma

**Figure 3:**
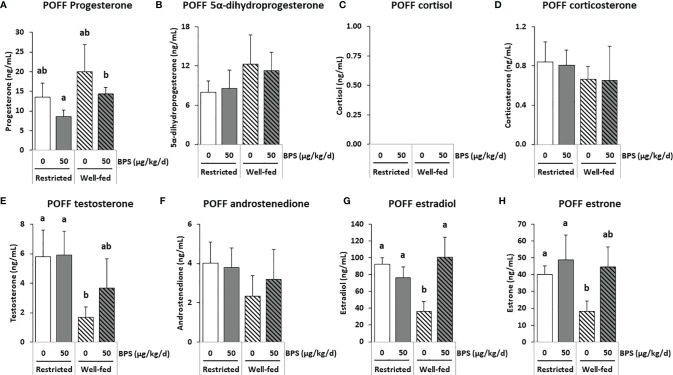
GC-MS/MS steroid hormones measurements in the preovulatory follicular fluid (POFF) of ewes according to their metabolic status and exposure to BPS. Concentrations of progestogens **(A, B)**, corticoids **(C, D)**, androgens **(E, F)** and estrogens **(G, H)** in pooled (3-4 ewes per pool) preovulatory follicular fluid (POFF) of ewes were assessed according to their metabolic status (restricted or well-fed) and previous dietary exposure to bisphenol S (0 or 50 µg/kg/day). Concentrations of cortisol **(C)** were not detectable in this biological fluid. Results are presented as mean +/- SEM (R0, n = 4; R50, n = 4; WF0, n = 6; WF50, n = 5). Bars with different letters indicate a significant difference (p < 0.05).

**Figure 4:**
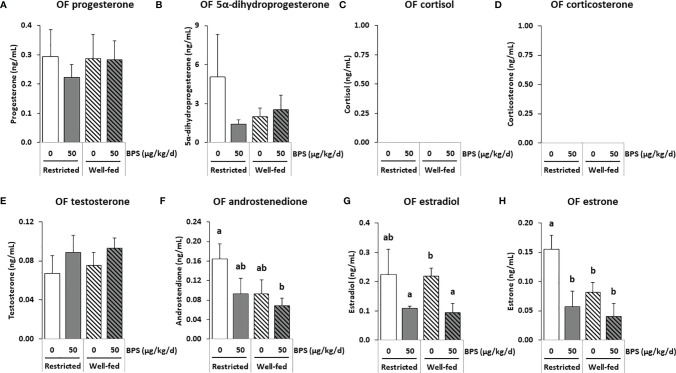
GC-MS/MS steroid hormones measurements in the oviductal fluid (OF) of ewes according to their metabolic status and exposure to BPS. Concentrations of progestogens **(A, B)**, corticoids **(C, D)** androgens **(E, F)** and estrogens **(G, H)** in pooled (3-4 ewes per pool) oviduct fluid (OF) of ewes were assessed according to their metabolic status (restricted or well-fed) and previous dietary exposure to bisphenol S (0 or 50 µg/kg/day). Concentrations of corticoids **(C, D)** were not detectable in this biological fluid. Results are presented as mean +/- SEM (R0, n = 4; R50, n = 4; WF0, n = 6; WF50, n = 5). Bars with different letters indicate a significant difference (p < 0.05).

**Figure 5:**
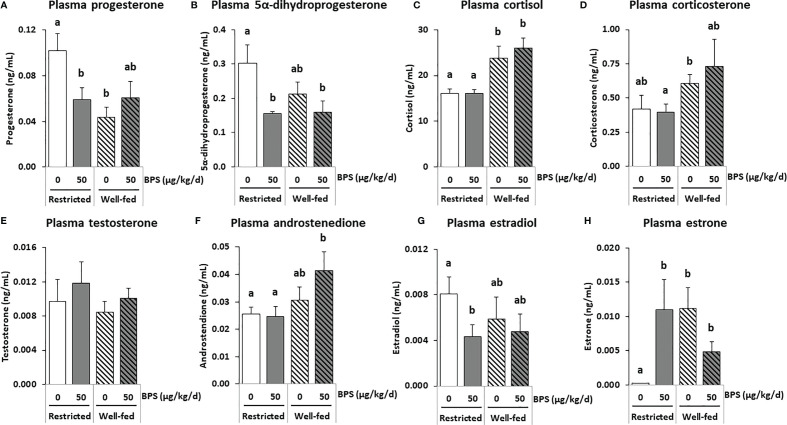
GC-MS/MS steroid hormones measurements in the plasma of ewes according to their metabolic status and exposure to BPS. Concentrations of progestogens **(A, B)**, corticoids **(C, D)**, androgens **(E, F)** and estrogens **(G, H)** in pooled (3-4 ewes per pool) circulating plasma of ewes were assessed according to their metabolic status (restricted or well-fed) and previous dietary exposure to bisphenol S (0 or 50 µg/kg/day). Results are presented as mean +/- SEM (R0, n = 4; R50, n = 4; WF0, n = 6; WF50, n = 5). Bars with different letters indicate a significant difference (p < 0.05).

**Figure 6:**
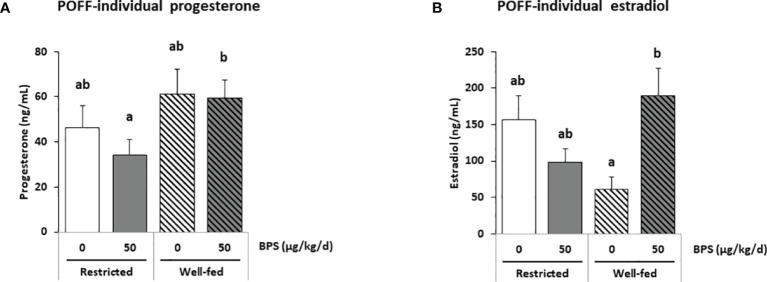
ELISA steroid hormones measurements in individual preovulatory follicular fluid (POFF) of ewes according to their metabolic status and exposure to BPS. Concentrations of progesterone **(A)** and estradiol **(B)** in individual preovulatory follicular fluid (POFF) of ewes were assessed according to their metabolic status (restricted or well-fed) and dietary exposure to bisphenol S (0 or 50 µg/kg/day). Results are presented as mean +/- SEM (R0, n = 18; R50, n = 19; WF0, n = 20; WF50, n = 18). Bars with different letters indicate a significant difference (p < 0.05).

A total of 36 steroids were detected, including 29 in preovulatory follicular fluid, 13 in oviduct fluid and 24 in plasma (**Tables 3**–**5**). Considering all compartments, 25 steroids were differentially abundant between the experimental groups. The main progestogens (progesterone and 5α-DHP), androgens (testosterone and androstenedione), estrogens (estradiol and estrone) and corticoids (cortisol and corticosterone) with significant differences between experimental groups in at least one compartment are shown in **Figures 3**–**5**. Additionally, concentrations of progesterone and estradiol in individual ewe preovulatory follicular fluid are shown in **Figure 6**.

**Table 3:** Steroid concentrations determined by GC/MS/MS in the preovulatory follicular fluid of ewes according to their metabolic status and dietary exposure to BPS.

Steroids	POOL SAMPLES						
	Mean concentration (ng/mL) ± SEM				p-value		
	R0	R50	WF0	WF50	Diet effect	BPS effect	Diet x BPS effect
**Precursors**	** **			** **			
PREG	194.800 ± 61.869	103.200 ± 28.385	124.467 ± 24.461	153.460 ± 7.076	0.76	0.37	0.072
20α-DHPREG	< 0.050	< 0.050	< 0.050	< 0.050	NA	NA	NA
17α-OH PREG	38.417 ± 9.359**^a^**	20.597 ± 4.952**^ab^**	11.145 ± 5.073**^b^**	23.330 ± 5.057**^ab^**	0.07	0.65	**0.028**
**Progestogens (C21)**							
Progesterone	13.550 ± 3.580**^ab^**	8.517 ± 1.763**^a^**	20.013 ± 6.793**^ab^**	14.398 ± 1.632**^b^**	0.25	0.34	0.99
5α-DHP	7.950 ± 1.748	8.590 ± 2.758	12.310 ± 4.412	11.302 ± 2.827	0.35	0.94	0.81
5β-DHP	0.177 ± 0.125	0.178 ± 0.132	0.113 ± 0.084	0.171 ± 0.088	0.75	0.64	0.73
20α-DHP	2.677 ± 0.737	1.315 ± 0.397	2.977 ± 0.794	2.526 ± 0.245	0.28	0.17	0.51
3α5α-THP	6.902 ± 1.000	5.052 ± 2.015	5.263 ± 1.096	3.732 ± 1.770	0.33	0.27	0.96
3α5β-THP	1.222 ± 0.738	1.105 ± 0.389	1.568 ± 0.575	1.046 ± 0.444	0.80	0.59	0.75
3β5α-THP	0.294 ± 0.098	0.574 ± 0.410	0.747 ± 0.448	1.053 ± 0.875	0.48	0.64	0.99
3α5α20α-HHP	< 0.010	< 0.010	< 0.010	< 0.010	NA	NA	NA
3α5β20α-HHP	0.131 ± 0.043**^a^**	0.111 ± 0.051**^ab^**	0.086 ± 0.008**^ab^**	0.048 ± 0.024**^b^**	0.11	0.38	0.75
3α5β20β-HHP	< 0.200	< 0.200	< 0.200	< 0.200	NA	NA	NA
3β5α20β-HHP	0.684 ± 0.307	1.723 ± 1.247	1.181 ± 0.838	2.867 ± 2.744	0.77	0.52	0.88
3β5α20α-HHP	0.165 ± 0.078	0.609 ± 0.377	0.531 ± 0.340	0.466 ± 0.336	0.76	0.63	0.43
17α-OHP	37.185 ± 7.076**^a^**	22.625 ± 4.275**^b^**	27.062 ± 6.900**^ab^**	26.688 ± 2.748**^ab^**	0.62	0.22	0.24
16α-OHP	11.582 ± 3.468**^ab^**	11.390 ± 1.994**^a^**	6.346 ± 1.204**^b^**	7.668 ± 1.463**^ab^**	**0.040**	0.80	0.71
**Androgens (C19)**							
Testosterone	5.800 ± 1.783**^a^**	5.897 ± 1.628**^a^**	1.690 ± 0.689**^b^**	3.658 ± 1.010**^ab^**	0.060	0.52	0.54
5α-DHT	0.200 ± 0.056**^a^**	0.053 ± 0.016**^b^**	0.076 ± 0.016**^b^**	0.084 ± 0.008**^b^**	0.08	**0.004**	**0.001**
5β-DHT	0.239 ± 0.114**^a^**	1.150 ± 0.298**^b^**	0.855 ± 0.308**^ab^**	0.707 ± 0.164**^b^**	0.76	0.16	0.057
DHEA	3.760 ± 1.157	3.265 ± 0.261	1.247 ± 0.802	2.422 ± 0.959	0.08	0.72	0.37
ADIOL	2.335 ± 0.436**^a^**	2.070 ± 0.426**^a^**	0.465 ± 0.265**^b^**	1.838 ± 0.826**^ab^**	0.07	0.33	0.15
ADIONE	4.027 ± 1.068	3.787 ± 0.989	2.323 ± 1.049	3.202 ± 1.505	0.37	0.77	0.64
5α-DHADIONE	0.154 ± 0.107	0.106 ± 0.026	0.083 ± 0.023	0.101 ± 0.013	0.55	0.88	0.65
**Estrogens (C18)**							
Estradiol	92.270 ± 7.836**^a^**	76.205 ± 12.855**^a^**	36.427 ± 11.181**^b^**	100.340 ± 24.113**^a^**	0.34	0.16	**0.020**
E1	40.110 ± 5.168**^a^**	46.685 ± 14.805**^a^**	18.360 ± 6.138**^b^**	44.520 ± 11.877**^ab^**	0.21	0.10	0.39
E3	0.410 ± 0.077**^ab^**	0.445 ± 0.032**^a^**	0.300 ± 0.061**^b^**	0.356 ± 0.090**^ab^**	0.19	0.55	0.88
2-ME2	0.095 ± 0.033	0.171 ± 0.039	0.097 ± 0.048	0.088 ± 0.043	0.36	0.46	0.37
2-OH-E2	9.242 ± 2.515	10.730 ± 0.782	4.630 ± 2.183	6.924 ± 3.109	0.10	0.46	0.88
**Corticoids (C21)**							
**Glucocorticoids**							
Cortisol (F)	< 2.000	< 2.000	< 2.000	< 2.000	NA	NA	NA
Cortisone	< 2.000	< 2.000	< 2.000	< 2.000	NA	NA	NA
11-deoxyF	< 2.000	< 2.000	< 2.000	< 2.000	NA	NA	NA
3α5β-THF	< 2.000	< 2.000	< 2.000	< 2.000	NA	NA	NA
**Mineralocorticoids**							
Corticosterone (B)	0.842 ± 0.200	0.805 ± 0.158	0.662 ± 0.136	0.650 ± 0.348	0.5	0.92	0.99
11-dehydroB	1.050 ± 0.313**^a^**	0.250 ± 0.161**^b^**	0.822 ± 0.383**^ab^**	0.247 ± 0.124**^b^**	0.76	**0.027**	0.69
3α5α-THDOC	0.036 ± 0.016**^ab^**	0.022 ± 0.014**^a^**	0.070 ± 0.018**^ab^**	0.088 ± 0.016**^b^**	**0.013**	0.88	0.36

Ewes were either restricted (R groups) or well-fed (WF groups) and received BPS 0 or 50 µg/kg/day, for at least three months before the start of plasma/fluid collection. Thus, four groups were formed: R0 (n = 4), R50 (n = 4), WF0 (n = 6) and WF50 (n = 5). Results are presented as mean (ng/mL) ± SEM. Tukey post-tests are indicated by superscript letters, different letters indicating significantly different conditions (p < 0.05). Two-way ANOVA p-values are presented for the effects of diet, dietary exposure to BPS and the interaction of these effects. Bold text indicates significant differences (p < 0.05). See **Table 1** for the steroid abbreviations. NA, not available.

**Table 4:** Steroid concentrations determined by GC/MS/MS in the oviduct fluid of ewes according to their metabolic status and dietary exposure to BPS.

Steroids	POOL SAMPLES						
	Mean concentration (ng/mL) ± SEM				p-value		
	R0	R50	WF0	WF50	Diet effect	BPS effect	Diet x BPS effect
**Precursors**					** **		
PREG	1.013 ± 0.405	0.486 ± 0.285	0.620 ± 0.205	0.594 ± 0.082	0.567	0.286	0.322
20α-DHPREG	0.121 ± 0.033	0.140 ± 0.066	0.112 ± 0.031	0.073 ± 0.024	0.340	0.840	0.480
17α-OH PREG	NA	NA	NA	NA	NA	NA	NA
**Progestogens (C21)**							
Progesterone	0.295 ± 0.091	0.223 ± 0.044	0.286 ± 0.083	0.284 ± 0.063	0.744	0.653	0.668
5α-DHP	5.045 ± 3.285	1.417 ± 0.321	2.013 ± 0.630	2.524 ± 1.115	0.687	0.406	0.208
5β-DHP	NA	NA	NA	NA	NA	NA	NA
20α-DHP	< 0.010	< 0.010	< 0.010	< 0.010	NA	NA	NA
3α5α-THP	< 0.050	< 0.050	< 0.050	< 0.050	NA	NA	NA
3α5β-THP	< 0.050	< 0.050	< 0.050	< 0.050	NA	NA	NA
3β5α-THP	0.793 ± 0.707**^ab^**	0.061 ± 0.013**^a^**	0.267 ± 0.095**^ab^**	0.472 ± 0.233**^b^**	0.894	0.524	0.170
3α5α20α-HHP	0.049 ± 0.022	0.021 ± 0.016	0.034 ± 0.010	0.025 ± 0.009	0.621	0.173	0.569
3α5β20α-HHP	0.131 ± 0.051**^ab^**	0.231 ± 0.063**^a^**	0.103 ± 0.033**^b^**	0.050 ± 0.028**^b^**	**0.025**	0.624	0.097
3α5β20β-HHP	NA	NA	NA	NA	NA	NA	NA
3β5α20β-HHP	0.908 ± 0.858**^ab^**	0.097 ± 0.048**^a^**	0.334 ± 0.120**^b^**	0.262 ± 0.141**^ab^**	0.843	0.272	0.460
3β5α20α-HHP	0.630 ± 0.543	0.094 ± 0.036	0.195 ± 0.078	0.289 ± 0.172	0.766	0.514	0.242
17α-OHP	NA	NA	NA	NA	NA	NA	NA
16α-OHP	NA	NA	NA	NA	NA	NA	NA
**Androgens (C19)**							
Testosterone	0.067 ± 0.019	0.089 ± 0.018	0.075 ± 0.013	0.093 ± 0.010	0.667	0.194	0.868
5α-DHT	NA	NA	NA	NA	NA	NA	NA
5β-DHT	NA	NA	NA	NA	NA	NA	NA
DHEA	< 0.010	< 0.010	< 0.010	< 0.010	NA	NA	NA
ADIOL	< 0.010	< 0.010	< 0.010	< 0.010	NA	NA	NA
ADIONE	0.165 ± 0.030**^a^**	0.093 ± 0.031**^ab^**	0.092 ± 0.029**^ab^**	0.069 ± 0.015**^b^**	0.102	0.100	0.395
5α-DHADIONE	NA	NA	NA	NA	NA	NA	NA
**Estrogens (C18)**							
Estradiol	0.224 ± 0.086**^ab^**	0.109 ± 0.006**^a^**	0.218 ± 0.028**^b^**	0.094 ± 0.030**^a^**	0.837	**0.007**	0.927
E1	0.155 ± 0.024**^a^**	0.057 ± 0.026**^b^**	0.082 ± 0.017**^b^**	0.041 ± 0.022**^b^**	0.061	**0.007**	0.214
E3	< 0.050	< 0.050	< 0.050	< 0.050	NA	NA	NA
2-ME2	< 0.010	< 0.010	< 0.010	< 0.010	NA	NA	NA
2-OH-E2	NA	NA	NA	NA	NA	NA	NA
**Corticoids (C21)**							
**Glucocorticoids**							
Cortisol (F)	< 2.000	< 2.000	< 2.000	< 2.000	NA	NA	NA
Cortisone	< 5.000	< 5.000	< 5.000	< 5.000	NA	NA	NA
11-deoxyF	< 0.100	< 0.100	< 0.100	< 0.100	NA	NA	NA
3α5β-THF	< 0.500	< 0.500	< 0.500	< 0.500	NA	NA	NA
**Mineralocorticoids**							
Corticosterone (B)	< 0.500	< 0.500	< 0.500	< 0.500	NA	NA	NA
11-dehydroB	< 0.010	< 0.010	< 0.010	< 0.010	NA	NA	NA
3α5α-THDOC	NA	NA	NA	NA	NA	NA	NA

Ewes were either restricted (R groups) or well-fed (WF groups) and received BPS 0 or 50 µg/kg/day, for at least three months before the start of plasma/fluid collection. Thus, four groups were formed: R0 (n = 4), R50 (n = 4), WF0 (n = 6) and WF50 (n = 5). Results are presented as mean (ng/mL) ± SEM. Tukey post-tests are indicated by superscript letters, different letters indicating significantly different conditions (p < 0.05). Two-way ANOVA p-values are presented for the effects of diet, dietary exposure to BPS and the interaction of these effects. Bold text indicates significant differences (p < 0.05). See **Table 1** for the steroid abbreviations. NA, not available.

**Table 5:** Steroid concentrations determined by GC/MS/MS in the plasma of ewes according to their metabolic status and dietary exposure to BPS.

Steroids	POOL SAMPLES						
	Mean concentration (ng/mL) ± SEM				p-value		
	R0	R50	WF0	WF50	Diet effect	BPS effect	Diet x BPS effect
**Precursors**	** **			** **			
PREG	2.210 ± 0.522**^ab^**	1.857 ± 0.276**^a^**	2.240 ± 0.162**^a^**	3.284 ± 0.451**^b^**	0.055	0.375	0.065
20α-DHPREG	0.045 ± 0.009**^a^**	0.047 ± 0.007**^ab^**	0.051 ± 0.012**^ab^**	0.079 ± 0.012**^b^**	0.122	0.209	0.300
17α-OH PREG	NA	NA	NA	NA	NA	NA	NA
**Progestogens (C21)**							
Progesterone	0.102 ± 0.015**^a^**	0.059 ± 0.011**^b^**	0.044 ± 0.009**^b^**	0.061 ± 0.014**^ab^**	**0.040**	0.315	**0.030**
5α-DHP	0.302 ± 0.053**^a^**	0.155 ± 0.006**^b^**	0.212 ± 0.035**^ab^**	0.159 ± 0.033**^b^**	0.264	**0.018**	0.233
5β-DHP	NA	NA	NA	NA	NA	NA	NA
20α-DHP	0.017 ± 0.003	0.017 ± 0.005	0.021 ± 0.005	0.029 ± 0.007	0.149	0.423	0.489
3α5α-THP	1.259 ± 0.150**^a^**	0.718 ± 0.201**^a^**	0.217 ± 0.039**^b^**	0.167 ± 0.041**^b^**	**< 0.001**	**0.016**	**0.042**
3α5β-THP	0.097 ± 0.032	0.143 ± 0.052	0.123 ± 0.020	0.126 ± 0.032	0.933	0.472	0.554
3β5α-THP	0.004 ± 0.004**^a^**	0.011 ± 0.005**^ab^**	0.015 ± 0.004**^b^**	0.012 ± 0.003**^ab^**	0.144	0.669	0.263
3α5α20α-HHP	0.029 ± 0.009	0.045 ± 0.011	0.030 ± 0.007	0.030 ± 0.006	0.399	0.353	0.350
3α5β20α-HHP	0.151 ± 0.025**^a^**	0.171 ± 0.043**^ab^**	0.101 ± 0.014**^ab^**	0.090 ± 0.005**^b^**	**0.010**	0.856	0.507
3α5β20β-HHP	0.047 ± 0.005**^a^**	0.052 ± 0.007**^a^**	0.026 ± 0.007**^b^**	0.030 ± 0.004**^b^**	**0.003**	0.484	1.000
3β5α20β-HHP	NA	NA	NA	NA	NA	NA	NA
3β5α20α-HHP	0.011 ± 0.003	0.009 ± 0.006	0.018 ± 0.004	0.012 ± 0.002	0.202	0.332	0.656
17α-OHP	NA	NA	NA	NA	NA	NA	NA
16α-OHP	NA	NA	NA	NA	NA	NA	NA
**Androgens (C19)**							
Testosterone	0.010 ± 0.003	0.012 ± 0.002	0.008 ± 0.001	0.010 ± 0.001	0.421	0.321	0.880
5α-DHT	NA	NA	NA	NA	NA	NA	NA
5β-DHT	NA	NA	NA	NA	NA	NA	NA
DHEA	0.015 ± 0.005	0.013 ± 0.002	0.011 ± 0.003	0.012 ± 0.004	0.496	0.935	0.740
ADIOL	0.016 ± 0.001	0.028 ± 0.010	0.020 ± 0.004	0.017 ± 0.003	0.512	0.498	0.154
ADIONE	0.025 ± 0.002**^a^**	0.025 ± 0.004**^a^**	0.031 ± 0.005**^ab^**	0.041 ± 0.007**^b^**	**0.046**	0.349	0.283
5α-DHADIONE	NA	NA	NA	NA	NA	NA	NA
**Estrogens (C18)**							
Estradiol	0.008 ± 0.001**^a^**	0.004 ± 0.001**^b^**	0.006 ± 0.002**^ab^**	0.005 ± 0.002**^ab^**	0.612	0.165	0.470
E1	< 0.0005**^a^**	0.011 ± 0.004**^b^**	0.011 ± 0.003**^b^**	0.005 ± 0.001**^b^**	0.417	0.455	**0.007**
E3	< 0.001	< 0.001	< 0.001	< 0.001	NA	NA	NA
2-ME2	< 0.005	< 0.005	< 0.005	< 0.005	NA	NA	NA
2-OH-E2	NA	NA	NA	NA	NA	NA	NA
**Corticoids (C21)**							
**Glucocorticoids**							
Cortisol (F)	16.052 ± 1.002**^a^**	16.130 ± 0.842**^a^**	23.735 ± 2.659**^b^**	26.040 ± 2.217**^b^**	**0.002**	0.609	0.605
Cortisone	10.775 ± 3.209	24.217 ± 8.293	29.593 ± 7.679	12.750 ± 6.289	0.605	0.794	0.052
11-deoxyF	0.149 ± 0.022	0.115 ± 0.047	0.106 ± 0.043	0.270 ± 0.096	0.409	0.326	0.130
3α5β-THF	0.257 ± 0.044**^a^**	0.243 ± 0.022**^a^**	0.271 ± 0.012**^a^**	0.382 ± 0.023**^b^**	**0.008**	0.070	**0.024**
**Mineralocorticoids**							
Corticosterone (B)	0.420 ± 0.101**^ab^**	0.397 ± 0.061**^a^**	0.608 ± 0.064**^b^**	0.731 ± 0.197**^ab^**	**0.033**	0.796	0.610
11-dehydroB	3.822 ± 0.674	2.767 ± 0.850	2.485 ± 0.525	20.048 ± 0.740	0.155	0.306	0.681
3α5α-THDOC	NA	NA	NA	NA	NA	NA	NA

Ewes were either restricted (R groups) or well-fed (WF groups) and received BPS 0 or 50 µg/kg/day, for at least three months before the start of plasma/fluid collection. Thus, four groups were formed: R0 (n = 4), R50 (n = 4), WF0 (n = 6) and WF50 (n = 5). Results are presented as mean (ng/mL) ± SEM. Tukey post-tests are indicated by superscript letters, different letters indicating significantly different conditions (p < 0.05). Two-way ANOVA p-values are presented for the effects of diet, dietary exposure to BPS and the interaction of these effects. Bold text indicates significant differences (p < 0.05). See **Table 1** for the steroid abbreviations. NA, not available.

#### 3.4.1 BPS Effects on Steroid Content in Preovulatory Follicular Fluid

In the preovulatory follicular fluid, estradiol (36-100 ng/mL) and estrone (18-47 ng/mL) were the most abundant estrogens. High concentrations of pregnenolone (103-195 ng/mL) and its metabolite 17α-hydroxypregnenolone, as well as the progestogens progesterone (9-20 ng/mL) and its hydroxylated metabolite 17α-hydroxyprogesterone were also measured (**Table 3** and **Supplementary Figure 1**). Among the corticoids, only mineralocorticoids were detected at concentrations below 1 ng/mL. The interaction between exposure to BPS and diet was significant for estradiol (p = 0.020), 17α-hydroxypregnenolone (p = 0.028) and the androgen 5α-dihydrotestosterone (p = 0.001; **Table 3**). When measuring estradiol in individual preovulatory follicular fluid, the interaction between BPS and diet was more significant than in pooled samples (p = 0.002, **Figure 6B**).

Exposure to BPS decreased the levels of 5α-dihydrotestosterone (p = 0.004) and of 11-dehydrocorticosterone (p = 0.027) in restricted ewes only (**Table 3**). The 2-way ANOVA post-tests also indicated that well-fed ewes exposed to BPS (WF50) had three-fold increased estradiol levels in the preovulatory follicular fluid compared to unexposed well-fed ewes (WF0); this effect was absent in restricted ewes (**Figure 3G**). The same pattern was found for estrone in well-fed ewes without statistical significance. In restricted ewes, post-tests indicated a decrease of 17α-hydroxyprogesterone level and an increase of 5β-dihydrotestosterone level after BPS exposure.

Overall, the well-fed diet decreased the concentration of 16α-hydroxyprogesterone and increased the concentration of 3α5α-tetrahydrodeoxycorticosterone compared to well-fed ewes (**Table 3**). Post-tests also revealed a decrease in 17α-hydroxypregnenolone, testosterone, 5a-dihydrotestosterone, androstenediol, estradiol and estrone in well-fed ewes (**Table 3**). In individual preovulatory follicular fluid, a diet effect was reported for progesterone (**Figure 6A**), with on average 1.4-fold more abundant in the preovulatory follicular fluid of well-fed ewes compared to restricted ewes. In addition, the progesterone level in follicular fluid was 1.7-fold higher in WF50 ewes compared to R50 ewes (**Figure 6A**).

#### 3.4.2 BPS Effects on Steroid Content in Oviduct Fluid

The concentration ranges were lower in the oviduct fluid as compared to the preovulatory follicular fluid. Estradiol and progesterone were measured at mean concentrations ranging from 0.1 to 0.3 ng/mL while androgens were below 0.1 ng/mL and corticoids were undetectable (**Table 4** and **Supplementary Figure 1**). No significant interaction between BPS exposure and the diet was found. BPS exposure decreased the concentrations of estradiol and of its precursor estrone in the oviduct fluid of ewes in both metabolic groups (p = 0.007; **Figures 4G, H**), and especially in well-fed ewes for the estradiol concentration (p < 0.001; **Figure 4G**). Progesterone levels were not affected by both BPS and diet status (**Figure 4A**). This was also the case for 5α dihydroprogesterone (**Figure 4B**), cortisol (**Figure 4C**), corticosterone (**Figure 4D**), testosterone (**Figure 4E**) and androstenedione (**Figure 4F**). The well-fed diet decreased the concentration of 3α5β20α-HHP (a metabolite of progesterone; p = 0.025) compared to well-fed ewes (**Table 4**). Post-tests indicated a decrease in estrone level in unexposed well-fed ewes and an increase in 3β5α-tetrahydroprogesterone and a decrease in 3α5β20α-hexahydroprogesterone in BPS-well-fed ewes.

#### 3.4.3 BPS Effects on Steroid Content in Plasma

In plasma, the most abundant steroid hormones were cortisol (16-26 ng/mL) and cortisone (11-30 ng/mL), while estradiol and progesterone concentrations ranged from 4-8 and 44-102 pg/mL, respectively (**Table 5** and **Supplementary figure 1**). A significant interaction between BPS exposure and diet was observed for progesterone (p = 0.030), 3α5α-tetrahydroprogesterone (p = 0.042), estrone (p = 0.007) and 3α5β-tetrahydrocortisol (p = 0.024; **Table 5**). A global BPS effect was observed for 5α-dihydroprogesterone (p = 0.018, **Figure 5B**) and 3α5α-tetrahydroprogesterone (p = 0.016, **Table 5**), with lower levels in the BPS-treated ewes compared to unexposed controls. Post-tests indicated that restricted ewes exposed to BPS (R50) displayed slightly lower circulating levels of progesterone (**Figure 5A**), 5α-dihydroprogesterone (**Figure 5B**) and estradiol (**Figure 5G**) compared to unexposed controls (R0), while estrone was increased (**Figure 5H**). Pregnenolone and 3α5β-tetrahydroprogesterone levels were also increased in BPS-treated well-fed ewes. The diet affected eight steroid concentrations including progesterone (p = 0.040, **Figure 5A**) and three progesterone metabolites (3α5α-THP, p < 0.001; 3α5β20α-HHP, p = 0.010; 3α5β20β-HHP, p = 0.003; **Table 5**) that decreased in the well-fed ewes compared to the restricted ewes. Conversely, the concentrations of androstenedione (p = 0.046; **Figure 5F**) and three corticosteroids, including cortisol (p = 0.002; **Figure 5C**) and corticosterone (p = 0.033; **Figure 5D**), increased in the plasma of well-fed ewes compared to restricted ewes. No effect was observed on testosterone (**Figure 5E**).

## 4 Discussion

To our knowledge, this study is the first to investigate the impact of a potential endocrine disruptor, BPS, on a wild range of secreted steroid hormones and their precursors and metabolites in mammals. Furthermore, this is the first study reporting and comparing these hormones in the plasma, follicular fluid and oviduct fluid at the same preovulatory period *in vivo*. The main outcome of this study is that dietary exposure to BPS at doses previously established as guidelines for BPA in Europe (50 µg/kg/day) had an effect on the concentration of estradiol in several biological fluids, and that this effect varied according to the metabolic status in ewes. Considering the three compartments investigated, all steroid pathways (progestogens, androgens, estrogens and corticoids) were affected by exposure to BPS and/or diet. In addition, our results evidenced a deleterious effect of a restricted diet on the ovarian weight, oviduct fluid volume and number of preovulatory follicles, which may have an impact on the reproductive capacity of ewes. These differences being observed when compared to the overfed group of ewes, further confirmation when compared to standard ewes is needed.

### 4.1 Absence of a Correlation in Steroid Profiling Between Compartments

In the present study, the absence of a correlation between the plasma, the follicular fluid and the oviduct fluid was observed for several steroid hormones. Indeed, while a reduction in progesterone was observed in the plasma of well-fed controls (WF0) compared to restricted controls (R0), no difference was observed in the follicular fluid. Even though the ovarian follicles are the primary source of progesterone, with the follicular fluid progesterone level being more than 100-fold higher compared with that in the oviduct fluid and plasma, similar patterns were not observed between compartments. Such an absence of correlation has been reported between plasma and seminal plasma in men (42). Because samples were collected during the preovulation period, plasma progesterone was at its lowest level, possibly explaining the lack of correlation between plasma and follicular levels. In the present study, regarding progesterone and estradiol levels, a steroidomic assay was performed on pooled samples (**Figure 3**) and an ELISA was performed on 75 individual preovulatory follicular fluids (**Figure 6**). These two assays provided consistent results, therefore, giving credit to the other steroids analyzed, meaning that the analyses on pooled samples likely reflect the analysis on individual samples. Moreover, the similar pattern between estradiol and estrone levels in follicular fluid also strengthened the data. It is notable that all steroids were measured after extraction, therefore, preventing a potential bias linked to steroid binding to albumin and lack of bioavailability. Additionally, the fold-reduction was not the same depending on the steroid between the follicular fluid and the other compartments in the present study. Indeed, considering estrogens (estradiol and estrone), they were between 400-900 fold lower in oviduct fluid and 1300-4000 fold lower in plasma compared to follicular fluid. Regarding androgens (testosterone and androstenedione), they were 30-50 fold lower in oviduct fluid and 100-400 fold lower in plasma compared to follicular fluid. On the contrary, progestogens showed greater variability. Indeed, while progesterone was 200 fold lower in oviduct fluid and 800 fold lower in plasma compared to follicular fluid, 5α-dihydroprogesterone was only 2-4 fold reduced in oviduct fluid and plasma compared to follicular fluid (**Supplementary Figure 1**). These differences between ovarian steroids suggest that passive diffusion is not the mechanism through which ovarian steroids reach the plasma compartment. It is, therefore, possible that BPS could impair this mechanism and contribute to the absence of correlation reported between compartments with the primary source of steroids. Further studies are required to decipher this discrepancy. Nevertheless, intrafollicular steroids would be more physiologically meaningful in terms of effect on the enclosed oocyte. Additional studies performing steroidomic assays on follicular fluid would, therefore, be helpful to highlight environmental factor effects on the endocrine environment and the quality of female gametes.

### 4.2 Effect of BPS or Diet on Progesterone and Corticoid Secretion

Regarding progesterone, previous studies have shown a lower level of progesterone in the circulating plasma of rats exposed to BPS (43). This is relevant to the decrease in plasma progesterone observed in restricted ewes after BPS exposure. An inhibitory effect of BPS on progesterone secretion has been reported in human (10 µM) and ovine (10 µM) granulosa cells *in vitro* (6, 12). Such a decrease in progesterone was observed but not significant in ewe follicular fluid *in vivo*. In the present study, ewes were chronically exposed to nanomolar BPS doses, whereas granulosa cells were exposed *in vitro* to acute micromolar BPS levels. Such differences in concentrations as well as in the species considered has led to varying results in the literature. Indeed, no effect of BPS on progesterone secretion was reported at a concentration lower than 100 µM in porcine or bovine granulosa cells (10, 11, 44). Moreover, some studies on BPA have already reported dose-dependent changes in progesterone secretion in both rat and porcine granulosa cells. Indeed, the opposite effects on granulosa cells were reported with either high concentrations (100 µM: inhibitory effect in rats) or lower concentrations (stimulatory effect: 100 nM in rats and 10 µM in pigs) (45, 46).

In the ovarian follicle, progesterone is critical to promoting the maturation and developmental capacity of the oocyte (44, 47), and low serum progesterone levels are associated with low ovulation percentages in women (48). In the oviduct, progesterone promotes sperm release from oviduct epithelial cells and the acquisition of fertilizing ability (49). In addition, progesterone has been reported to modulate sperm progressive motility, capacitation and the acrosome reaction in bulls and rams (50–52). Our data, therefore, suggest that the BPS-induced impairment in progesterone secretion in restricted ewes could be detrimental to oocyte quality and, consequently, fertility. This is in line with our previous results that suggest that the effects of BPS on oocyte quality assessed by *in vitro* embryo production after ovum pick-up also vary according to metabolic status (13).

Regarding the diet effect, in the present study, a decrease in preovulatory plasma progesterone was reported in well-fed controls, which is not in line with the reduction in plasma progesterone previously observed in low body condition score (BCS) ewes (25). Nevertheless, this reduction in plasma progesterone in low BCS ewes was observed during the off-season period, after the ram effect triggered the rise of progesterone levels. The progesterone level was, therefore, measured at a different physiological stage of the cycle that renders both experiments difficult to compare and that might explain the difference with the present study.

Concerning corticoids, an increase in plasma cortisol concentration was shown for well-fed ewes. These data are in agreement with the literature. Indeed, rabbits on a high calorie diet also had an increase in plasma cortisol concentration compared to rabbits on a standard diet (53). Cortisol, a glucocorticoid steroid produced by the adrenal glands, contributes to metabolic regulation as a catabolic hormone. Cortisol stimulates protein breakdown to release amino acids that can be used in hepatic gluconeogenesis to produce glucose, the breakdown of glycogen and the release of fatty acids from adipose depots (28). Cortisol also affects the developmental potential of the oocyte and embryo. The cortisol:cortisone ratio in the follicular fluid was previously reported to be positively correlated with the probability of conception subsequent to embryo transfer in women (54). Nevertheless, cortisol levels were high in plasma due to the process of culling the animals, which induced stress, therefore, masking potential BPS effects.

### 4.3 The Effect of BPS on Estradiol Varied According to the Compartment and Metabolic Status

Considering all compartments, seven steroids (estradiol E2, estrone E1, progesterone PROG, 3α5α tetrahydroprogesterone 3α5α-THP, 17α-hydroxypregnenolone 17α-OH PREG, 5α-dihydrotestosterone 5α-DHT and 3α5β-tetrahydrocortisol 3α5β-THF) had a significant interaction between BPS exposure and metabolic status, meaning that the effect of BPS depended on the metabolic status. An interaction between the effects of BPS and the metabolic status of the ewe has already been observed on the follicular population and oocyte competence in early embryonic development (13). In another study, altered placental architecture and increased fetal mortality were shown in mice exposed to phthalates, an endocrine disruptor, when they had a fatty diet, compared to mice with a standard diet (55). In this study, BPS exposure led to a significant increase in preovulatory follicular fluid estradiol only in well-fed ewes, while no difference was reported in restricted ewes. Such an increase in estradiol was previously reported in the culture medium of bovine and ovine granulosa cells after acute exposure to 100 µM and 10 µM BPS, respectively (11, 12). Even though the ewes were exposed to much lower BPS concentrations than µM concentrations (undetectable BPS levels, i.e. lower than the 2 nM detection limit, and 43.6-102.5 nM BPS-g detected in follicular fluids of ewes in the present study), this effect on estradiol was still observed after chronic exposure, corresponding to human exposure (6, 56). This effect is in accordance with the reported estrogenomimetic effect of bisphenols and their ability to bind to nuclear as well as membrane estrogen receptors, reported in ovarian and breast cell lines (9, 57–59). The increase in estradiol secretion observed in the preovulatory follicular fluid could be related to an increase in the expression and activity of aromatase (CYP19A1) in the ovary, or a reduction in its hepatic catabolism. CYP19A1 is an enzyme required for the conversion of androgens into estrogens in the ovarian granulosa. BPS has been reported to increase aromatase mRNA expression in a human breast cell line (60) and its activity in both human breast cell line and in zebrafish embryos (60, 61). However, BPS has also been reported to decrease estradiol secretion in human (50 µM) and porcine (1 µM) granulosa cells (6, 10). Surprisingly, estradiol mean levels were increased in the preovulatory follicular fluid while decreased in the oviduct fluid of well-fed ewes exposed to BPS (WF50) compared to well-fed control ewes (WF0). Estradiol was also decreased in the plasma of all ewes exposed to BPS, but this change was significant only in restricted ewes. This difference in the effect of BPS according to the compartment might be due to a tissue-specific effect of BPS on the synthesis and catabolism of steroids.

Of note, the synthesis of progesterone and estradiol and the expression of steroidogenic hormones including aromatase were reported in the oviduct tissue of sows (62) and mares (63), suggesting that the transfer of sex steroid hormones from the follicular fluid *via* the blood is not the only source of estradiol in the oviduct fluid. Such synthesis of steroid hormones has not been reported in the ovine oviduct to date; however, our data highlight the potential impact of estrogenomimetics like BPS on the oviduct hormonal environment. Steroid hormones have been reported as important regulators of sperm capacitation, acrosome reaction (both prerequisites for fertilization) and migration in the female reproductive tract (64), so decreased estradiol concentrations in the oviduct fluid may have functional implications. Indeed, incubation of ram sperm in capacitating conditions with estrogen receptor agonists resulted in an enhanced acrosome reaction, while a receptor antagonist prevented this effect (51). The deletion of estrogen receptor α (ERα) led to impaired fertilization in mice (65). Estradiol is also involved in the suppression of oviductal protease activity, which is required for both fertilization and preimplantation embryo development (65) and in the regulation of oviduct inflammation in ewes (66).

### 4.4 The Overweight Population May Be More Sensitive to the Effects of BPS

Similar to what was observed in the preovulatory follicular fluid, the effect of BPS on estradiol in the oviduct fluid was significant only in well-fed ewes. The variability of the bisphenol effect depending on the metabolic status of exposed mammals could contribute to explaining the discrepancies reported in the literature. Not taking into account the metabolic status of individuals could induce a bias in the results and potentially hide some harmful effects of bisphenol present in a subgroup of the population. It is already known that, in humans, urinary BPS concentrations are higher in obese adults compared to non-obese adults (67). However, in addition to the possible accumulation of the lipophilic molecules of bisphenols in biological matrices (68), higher exposure to bisphenol due to lifestyle and diet is likely. Indeed, healthier food, meaning plastic-free food packaging, is related to lower urinary bisphenol levels (69, 70). Therefore, in addition to being more exposed to bisphenols, obese people could be also more sensitive to their effects. This result suggests a potentially higher sensitivity to the effects of BPS in a subgroup of the population, which could have broad implications in terms of future regulations and recommendations regarding the use of bisphenols in the food industry.

## 5 Conclusion

Chronic exposure of adult ewes to BPS affected the steroidome of the preovulatory follicular fluid, oviductal fluid and plasma. These disturbances were not similar between fluids, indicating a specific effect of BPS depending on the compartment. Estradiol was probably the most altered steroid, highlighting the estrogenic effects of BPS. In addition, the metabolic status of the ewe modulated the effects of BPS, with fat ewes being more sensitive to the effects of this endocrine disruptor. For most of the steroids measured, the dataset presented in the present study is unique and therefore the comparison with literature can only be limited. Nevertheless, a number of steroid hormones and metabolites did vary in our study, suggesting broad actions of BPS on steroid pathways. In light of our data, BPS can be considered an endocrine disruptor, the use of which should be prohibited.

## Data Availability Statement

The original contributions presented in the study are included in the article/**Supplementary Material**. Further inquiries can be directed to the corresponding author.

## Ethics Statement

All experimental procedures were conducted in accordance with the European Directive 2010/63/EU on the protection of animals used for scientific purposes and approved by the French Ministry of National Education, Higher Education, Research and Innovation after ethical assessment by the local ethics committee. “Comité d’Ethique en Expérimentation Animale Val de Loire (CEEA VdL)” (protocols registered under APAFIS numbers 13965-2018042008519239v2 and 14014-2018030717477406v2).

## Author Contributions

OT performed experiments, analyzed the data and wrote the paper. PL and AP performed experiments and helped write the paper. AD, OL, and PP performed experiments. OL took care of the animals. AD, CV, M-EL, VM, and AB helped write the paper. MS-D performed experiments, analyzed the data and wrote the paper. SE conceived the study, performed experiments, analyzed the data and wrote the paper. All authors contributed to the article and approved the submitted version.

## Funding

This study was financially supported by the INRAE, “Centre Val-de-Loire” Region (BEMOL project, APR IR 2017-00117108; PERFECT project, APR IR 2021-00144784), the French National Research Agency (MAMBO, project ANR-18-CE34-0011-01) and the BioMedecine Agency (FertiPhenol Project 18AMP006).

## Conflict of Interest

The authors declare that the research was conducted in the absence of any commercial or financial relationships that could be construed as a potential conflict of interest.

## Publisher’s Note

All claims expressed in this article are solely those of the authors and do not necessarily represent those of their affiliated organizations, or those of the publisher, the editors and the reviewers. Any product that may be evaluated in this article, or claim that may be made by its manufacturer, is not guaranteed or endorsed by the publisher.
