# Supplementary material for: Bisphenol S Alters the Steroidome in the Preovulatory Follicle, Oviduct Fluid and Plasma in Ewes With Contrasted Metabolic Status
Source: Front Endocrinol (Lausanne). 2022 May 24;13:892213. doi: 10.3389/fendo.2022.892213 (PMC9172638; doi:10.3389/fendo.2022.892213)
Supplement: Supplementary file 1 [file DataSheet_1.pdf]

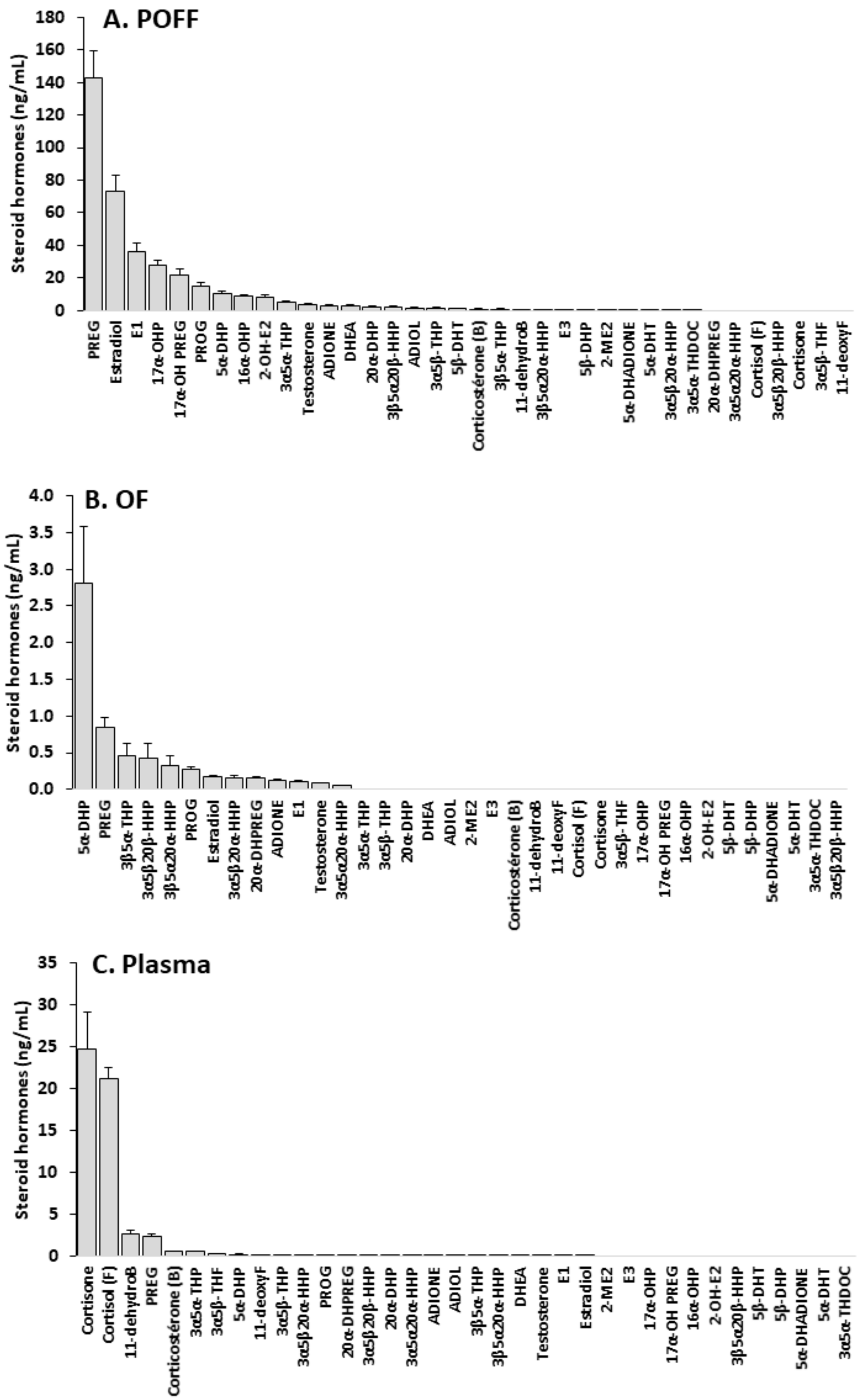

Supplementary Figure 1. Steroid concentrations in ovine preovulatory follicular fluid, oviduct fluid and plasma

The mean concentrations of each steroid hormone in all samples (n = 19) is reported for preovulatory follicular fluid (A), oviduct fluid (B) and plasma (C). Results are presented as mean +/- SEM.
